# Multisectoral approach to address Female Genital Mutilation: a case study from Portugal: Raquel Vareda

**DOI:** 10.1093/eurpub/ckac131.460

**Published:** 2022-10-25

**Authors:** R Vareda, J Valente, AM Alho, A Carmona, D Chaves, L Silva, S Ribeiro, M Paixão, A Leite

**Affiliations:** 1 Northern Lisbon Public Health Unit, Lisbon and Tagus Valley Regional Health Administration, Lisbon, Portugal; 2 Department of Public Health, Lisbon and Tagus Valley Regional Health Administration, Lisbon, Portugal; 3 António Luz Public Health Unit of Amadora, Lisbon and Tagus Valley Regional Health Administration, Lisbon, Portugal; 4 Arco Ribeirinho Public Health Unit, Lisbon and Tagus Valley Regional Health Administration, Lisbon, Portugal; 5 Sintra Public Health Unit, Lisbon and Tagus Valley Regional Health Administration, Lisbon, Portugal; 6 Central Lisbon Public Health Unit, Lisbon and Tagus Valley Regional Health Administration, Lisbon, Portugal; 7 Comprehensive Health Research Center, Universidade NOVA de Lisboa, Lisbon, Portugal; 8 Public Health Research Center, NOVA National School of Public Health, Universidade NOVA de Lisboa, Lisbon, Portugal

## Abstract

**Issue:**

Female genital mutilation (FGM) comprises all procedures that injury female genital organs for non-medical reasons, with several health impacts. Due to global migration, FGM has been increasingly recognised as a healthcare issue in Europe, affecting nearly 1 million women. In Portugal it is estimated that 5483 migrant women have undergone FGM in the Lisbon region. Intervention is required to tackle this issue.

**Description:**

Portugal launched the “Healthy Practices: End of FGM”, a multiagency project targeting Lisbon and Tagus Valley region. Project implementation started in Nov 2018 at 5 local public health units (PHU) and was scaled-up to 5 more in Feb 2020. Project comprises 3 main axes: 1) inclusion in public policy instruments; 2) professionals’ education and awareness; and 3) community intervention. We describe inclusion of FGM in public policy, professionals training and changes in FGM recording before and after intervention.

**Results:**

Between 2018-2022, inclusion of FGM in municipalities’ migration policies doubled. Between 2019-2021, 110 training sessions (n = 1722 professionals) were promoted. During pandemic years, only 344 (2020) and 202 (2021) were trained. Raising awareness and empowerment to risk communities happened mainly through local/online open sessions, workshops, flyer distribution, video projections. These occurred in all 10 PHU, mostly through partnerships with Non-Governmental Organizations and municipalities. According to the Portuguese Health Records, until 2018 there were only 300 women registered with FGM. Between 2019-2021, 363 more were added.

**Lessons:**

The multisectoral approach allowed PHU professionals to collaborate directly with external organizations from different society sectors. COVID-19 pandemic posed a challenge to implementation, especially in the community intervention axis. Notification numbers increased after interventions, though causality could not be established and impact evaluation is yet to be performed.

**Key messages:**

• Multisectoral projects for FGM intervention have specific implementation challenges, including how to justify and evaluate them, that must be considered in each setting.

• Training health professionals might increase identification and notification of FGM, but the impact in preventing FGM in the Portuguese reality is still largely unknown.

